# Effectively computing transition patterns with privacy-preserved trajectory datasets

**DOI:** 10.1371/journal.pone.0278744

**Published:** 2022-12-09

**Authors:** Jong Wook Kim, Beakcheol Jang

**Affiliations:** 1 Department of Computer Science, Sangmyung University, Seoul, Korea; 2 Graduate School of Information, Yonsei University, Seoul, Korea; Bu-Ali Sina University: Bu Ali Sina University, IRAN, ISLAMIC REPUBLIC OF

## Abstract

Recent advances in positioning techniques, along with the widespread use of mobile devices, make it easier to monitor and collect user trajectory information during their daily activities. An ever-growing abundance of data about trajectories of individual users paves the way for various applications that utilize user mobility information. One of the most common analysis tasks in these new applications is to extract the sequential transition patterns between two consecutive timestamps from a collection of trajectories. Such patterns have been widely exploited in diverse applications to predict and recommend next user locations based on the current position. Thus, in this paper, we explore the computation of the transition patterns, especially with a trajectory dataset collected using differential privacy, which is a de facto standard for privacy-preserving data collection and processing. Specifically, the proposed scheme relies on geo-indistinguishability, which is a variant of the well-known differential privacy, to collect trajectory data from users in a privacy-preserving manner, and exploits the functionality of the expectation-maximization algorithm to precisely estimate hidden transition patterns based on perturbed trajectory datasets collected under geo-indistinguishability. Experimental results using real trajectory datasets confirm that a good estimation of transition pattern can be achieved with the proposed method.

## Introduction

Recent advances in indoor and outdoor positioning techniques make it easier to monitor and collect user trajectory information during daily activities. An ever-growing abundance of data about trajectories of individual users paves the way for various applications [1–4]. However, similar to any other novel applications, these applications that leverage large-scale user trajectory data suffer from new problems as well. This is because the personal mobility information of individuals usually contains sensitive information, such as home and workplace addresses, hospital visit records, and political affiliation, which they do not want to disclose [5, 6]. Thus, most users are not comfortable to provide their trajectory information to those applications.

In the past decade, extensive studies have been conducted to protect user privacy when collecting and publishing trajectories, including spatial cloaking [7], mix-zone approach [8], and encryption-based scheme [9]. Recently, as differential privacy has emerged as a de facto standard for privacy-preserving data processing, there have been several efforts to apply differential privacy to the collection and publishing of trajectory data [10–13]. These privacy-preserving methods can alleviate, to some extent, users’ concerns about privacy exposure when providing their trajectory data to external service providers. However, from the service provider viewpoint, such privacy-preserving schemes lead to severe degradation in the utility of the collected dataset, eventually resulting in a loss in the quality of service.

One of the most common analysis tasks in applications that utilize user mobility information is to extract the sequential transition patterns between two consecutive timestamps, which corresponds to the probability that a user located at one location in the current timestamp moves to another location in the next timestamp. Such transition patterns have been widely exploited by diverse applications when predicting and recommending the next user location based on their current position. For example, the notion of first-order Markov chain, which models the sequential transition pattern between two consecutive point-of-interests (POIs), is used to recommend the next POI candidates to users in [2]. In [1], the sequential transition influences are integrated into the matrix factorization algorithm to enhance the accuracy of next location recommendations. In [14], transition probability matrices constructing from sequential rules are used to make location prediction for location-based-service users.

**Fig 1:**
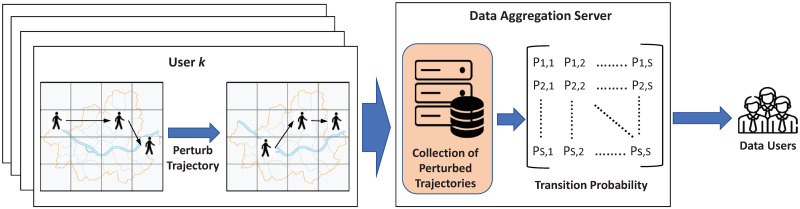
High level architecture of the proposed framework.

Computing transition probabilities from a collection of true trajectories is not difficult. However, the same cannot be said for the dataset consisting of privacy-preserved trajectory data. Let us consider a motivational example in Fig 1. As shown in the figure, users agree to contribute their trajectories to service providers for analysis purposes. However, owing to privacy concerns, they provide perturbed trajectories, which are obtained using geo-indistinguishability (GeoInd) [10] that is a variant of differential privacy, instead of true trajectories. During this process, it is guaranteed that individual users’ true location trajectories are not disclosed to the outside of the users’ devices, thus, protecting the location privacy of users. However, such a privacy-preserving mechanism leads to a loss in the utility of collected dataset. Subsequently, the resulting sequential transition patterns extracted from such dataset may have a lower utility.

To address this problem, in this paper, we explore the computation of the sequential transition patterns (i.e., transition probability between two locations) with a perturbed trajectory dataset collected using GeoInd. There have been several efforts to compute aggregate statistics from users’ trajectory datasets in a privacy-preserving manner using GeoInd. Most of these works are dedicated to the estimation of population density distribution, which corresponds to static information of users, at a specific timestamp. For example, Ren and Tang [15] presented a vehicle location privacy protection framework based on GeoInd to compute traffic density distribution at a specific timestamp. Wang *et al*. [16] and Qui *et al*. [17] presented mobile crowdsensing frameworks in which workers’ density distribution is computed in a privacy-preserving manner using GeoInd. On the contrary, our work aims to estimate users’ transition probabilities, which correspond to moving information of users, from perturbed trajectory datasets collected using GeoInd. To the best of our knowledge, this is the first attempt to estimate transition probability between two locations under GeoInd setting. The contributions of this paper can be briefly summarized as follows: We develop a privacy-preserving framework based on GeoInd for the computation of transition probability between two locations. In particular, the proposed scheme exploits the functionality of the expectation-maximization (EM) algorithm to precisely estimate hidden transition patterns based on perturbed trajectory dataset. Through experiments with real data, it is demonstrated that a good estimation of transition pattern can be achieved with the proposed framework.

## Background and problem definition

### Geo-indistinguishability

GeoInd is an extended version of differential privacy with a distance metric to provide a privacy-preserving mechanism for location data [10]. Formally, *ϵ*-GeoInd is defined as follows:

**Definition 1** (*ϵ*-GeoInd) Let X be a set of possible user locations and Y be a set of reported locations. It is commonly assumed that X is equal to Y. Let us assume that a randomized mechanism, *K*, probabilistically generates a perturbed location from a true location of a user. Then, *K* satisfies *ϵ*-GeoInd, if and only if for (1) all $x,x^{\prime }\in X$ and (2) any output location, y∈Y, the following equation is satisfied:

$$\begin{array}{c} K\left(x\right)\left(y\right)\leq e^{\epsilon \cdot d(x,x^{\prime })}\times K\left(x^{\prime }\right)\left(y\right), \end{array}$$ (1)

where *d*(*x*, *x*′) is the distance between *x* and *x*′.

The parameter *ϵ*, which corresponds to a privacy budget, determines a trade-off between the level of privacy and data utility. That is, smaller values of *ϵ* ensure a stronger privacy guarantee, introducing larger perturbation to the true location; whereas larger values of *ϵ* guarantee a weaker privacy but introduce smaller noise to the true location. This definition denotes that, given a reported location *y* that is an output of the randomized mechanism *K*, the ability of an adversary to identify whether the actual location of a user is *x* or *x*′ is limited by the privacy budget (i.e., *ϵ*) and distance between *x* and *x*′. This implies that the closer two locations are, the more indistinguishable they are.

### Problem definition

In this subsection, we introduce key notations and formally define the problem. The main notations used in the rest of this paper are summarized in Table 1.

**Table 1:** Summary of major notations.

Notation	Description
*TR*_*k*_	True trajectory of the *k*-th user
*TR*_*kp*_	Perturbed trajectory of the *k*-th user
*DB*	Collection of all pairs of perturbed locations
*G*	*m* × *n* grid
*g* ∈ *G*	True location
*g*′ ∈ *G*	Perturbed location
*OM*	Obfuscation matrix
*OM*[*x*, *y*]	Prob. of perturbing *g*_*x*_ to $g_{y}^{\prime }$ by *OM*
*p*(*g*_*x*_)	Prob. that a user’s location is *g*_*x*_
$p(g_{y}^{\prime }\;|\;g_{x})$	Prob. that $g_{y}^{\prime }$ is randomly generated from *g*_*x*_
$p(g_{x}\;|\;g_{y}^{\prime })$	Prob. that a true location of $g_{y}^{\prime }$ is *g*_*x*_

Let us assume that the entire area is partitioned into *m* × *n* grid *G*. Then, if the current location of a specific user belongs to a grid *g* ∈ *G* at time *t*, the location of this user is represented as (*g*, *t*). The trajectory of the *k*-th user over *r* timestamps is represented as the sequence of *r* locations, such as *TR*_*k*_ = {(*g*_*k*1_, *t*_*k*1_), (*g*_*k*2_, *t*_*k*2_), …, (*g*_*kr*_, *t*_*kr*_)}. Given the *k*-th user’s trajectory *TR*_*k*_, let $TR_{kp}=\left\{\left(g_{k1p}^{\prime },t_{k1}\right),\left(g_{k2p}^{\prime },t_{k2}\right),\ldots ,\left(g_{krp}^{\prime },t_{kr}\right)\right\}$ be the corresponding perturbed trajectory obtained by the perturbation mechanism of GeoInd. Here, $g_{ip}^{\prime }$ corresponds to the perturbed location generated from the *i*-th true location *g*_*i*_ using GeoInd. Here, to differentiate between the perturbed and true locations, we use *g* to represent a true location and *g*′ to represent a perturbed location. We will also omit the subscript, *k*, if it is clear from context.

Let $DB_{TR_{p}}=\left\{TR_{1p},TR_{2p},\ldots TR_{fp}\right\}$ be the collection of perturbed trajectories received from all users that are maintained by the data aggregation server. Here, *f* is the number of users. For each $TR_{kp}=\left\{\left(g_{1p}^{\prime },t_{1}\right),\left(g_{2p}^{\prime },t_{2}\right),\ldots ,\left(g_{rp}^{\prime },t_{r}\right)\right\}$ in *DB*, we can extract a set of perturbed location pairs in two adjacent timestamps, such as $TR_{kp\_pair}=\left\{\left(g_{1p}^{\prime },g_{2p}^{\prime }\right),\left(g_{2p}^{\prime },g_{3p}^{\prime }\right),\ldots ,\left(g_{(r-1)p}^{\prime },g_{rp}^{\prime }\right)\right\}$. Then, let *DB* be a collection of all such pairs of locations that can be obtained from all perturbed trajectories in $DB_{TR_{p}}$. That is, *DB* is formally defined as follows:

$$\begin{array}{c} DB=\underset{TR_{kp}\in DB_{TR_{p}}}{⋃}TR_{kp\_pair}, \end{array}$$ (2)

where ⋃ represents the union all operator which allows duplicates.

Let *p*(*g*_*x*_ → *g*_*y*_) be the transition probability from *g*_*x*_ to *g*_*y*_, which denotes the probability that a user currently located at *g*_*x*_ moves to *g*_*y*_ in the next timestamp. Given *DB*, a straightforward solution for obtaining transition probabilities is to directly compute *p*(*g*_*x*_ → *g*_*y*_) based on the perturbed trajectories, such as

$$\begin{array}{c} p\left(g_{x}\to g_{y}\right)=\frac{cnt(\left(g_{x}^{\prime },g_{y}^{\prime }\right),DB)}{\sum_{g_{z}\in G}cnt(\left(g_{x}^{\prime },g_{z}^{\prime }\right),DB)}, \end{array}$$ (3)

where $cnt(\left(g_{x}^{\prime },g_{y}^{\prime }\right),DB)$ denotes the number of times that the location pair $\left(g_{x}^{\prime },g_{y}^{\prime }\right)$ appears in *DB*. However, this straightforward approach cannot accurately compute the transition probabilities because it does not consider the effect of the location perturbation mechanism of GeoInd. Thus, in this study, we aim to propose a novel scheme to accurately estimate the transition probabilities for all pairs of locations in *G* with the collection of perturbed trajectories.

## Privacy-preserving computation of transition probability between locations

In this section, we describe our privacy-preserving framework for effectively computing transition probabilities based on the collection of perturbed trajectories collected using GeoInd.

### Privacy-preserving trajectory collection

There are two mechanisms to achieve *ϵ*-GeoInd: the Laplace and optimization mechanisms. The Laplace mechanism is simple but it is known to introduce large noise to the collected data, resulting in a lower utility. On the contrary, the optimization mechanism can provide the maximum utility for the collected data, while satisfying *ϵ*-GeoInd. In the optimization mechanism of GeoInd, the server first computes the obfuscation matrix, *OM*, by solving a linear programming problem. Let *d*(⋅, ⋅) be the distance metric between two grids in *G*, such as the Euclidean distance or the Manhattan distance. Let us further assume that *κ*_*G*_ be the prior probability distribution on users’ possible locations. *κ*_*G*_ can be either defined by a uniform distribution or inferred from the distribution of available historical data. Then, the obfuscation matrix *OM* can be obtained by solving the following linear programming problem [18, 19]:

$$\begin{array}{c} \begin{array}{ccc} min:\;\; & \sum_{g_{u},g_{v}\in G}\kappa_{G}\left(g_{u}\right)\cdot OM\left[u,v\right]\cdot d\left(g_{u},g_{v}^{\prime }\right) & \\ s.t.:\;\; & OM\left[u,v\right]\leq e^{\epsilon \cdot d(g_{u},g_{w})}\times OM\left[w,v\right] & g_{u},g_{w},g_{v}^{\prime }\in G\\ & \sum_{g_{v}^{\prime }\in G}OM\left[u,v\right]=1 & g_{u}\in G\\ & OM[u,v]\geq 0 & g_{u},g_{v}^{\prime }\in G \end{array} \end{array}$$ (4)

Here, *OM*[*u*, *v*] represents the probability that a perturbed location $g_{v}^{\prime }$ is randomly generated from a true location *g*_*u*_. The first constraint corresponds to the definition of GeoInd. The second constraint $\sum_{g_{v}^{\prime }\in G}OM\left[u,v\right]=1$ denotes that the sum of probabilities that each location is perturbed to other locations should be 1. The third constraint *OM*[*u*, *v*]≥0 denotes that the probability is no less than 0. It is well known that the number of constraints of the abovementioned linear optimization problem is proportional to $O(|G{|}^{3})$; therefore, the computational overhead of the optimal mechanism is significant. To alleviate the computational overhead of the optimal mechanism, several studies based on an approximation technique have been conducted in the literature [18, 19].

After computing the obfuscation matrix *OM*, the server distributes it to each user. Once receiving *OM*, each user perturbs his/her true trajectory according to the probabilities encoded in *OM*. Let us focus on the *k*-th user’s trajectory *TR*_*k*_ = {(*g*_1_, *t*_1_), (*g*_2_, *t*_2_), …, (*g*_*r*_, *t*_*r*_)}. Given the *k*-th user’s trajectory *TR*_*k*_, the corresponding perturbed trajectory $TR_{kp}=\left\{\left(g_{1p}^{\prime },t_{1}\right),\left(g_{2p}^{\prime },t_{2}\right),\ldots ,\left(g_{rp}^{\prime },t_{r}\right)\right\}$ is computed in a way that the perturbed location $g_{ip}^{\prime }$ is randomly generated from the true location *g*_*i*_ based on the probabilities encoded in *OM*. Finally, the perturbed trajectory, *TR*_*kp*_, is sent to the data aggregation server. Note that during this process, true locations along users’ trajectories are not exposed to the outside of their devices, because the trajectory perturbation is performed on their devices.

### Computation of transition probability with a collection of perturbed trajectories

In this subsection, we present the proposed method that effectively computes the transition probability between pairs of locations in *G* based on a collection of perturbed trajectories, which are collected under *ϵ*-GeoInd. Given *DB*, we first compute the joint probability *P*(*g*_*x*_, *g*_*y*_), which denotes the probability that a user’s current location is *g*_*x*_ and next location is *g*_*y*_, by leveraging the functionality of the EM algorithm. We next compute the transition probability, *p*(*g*_*x*_ → *g*_*y*_), using the joint probability *P*(*g*_*x*_, *g*_*y*_).

EM is an iterative algorithm that sequentially runs two steps, namely, E-step (expectation) and M-step (maximization). In the E-step, the expected value of the likelihood is computed based on the current parameters and observed variables, whereas in the M-step, an estimation on the parameters is performed to maximize the likelihood function. To leverage the EM algorithm, we first need to define a likelihood function. The collection of location pairs, *DB*, can be viewed as the set of observed variables, *O* = {*o*_1_, *o*_2_, …, *o*_|*DB*|_}, where the *i*-th observed variable *o*_*i*_ corresponds to the *i*-th perturbed location pair $(g_{cur\_i}^{\prime },g_{nex\_i}^{\prime })\in DB$. We also introduce the set of latent variables *Z* = {*z*_1_, *z*_2_, …, *z*_|*DB*|_} where *z*_*i*_, which is associated to *o*_*i*_, is the *mn* × *mn* matrix. Here, *z*_*i*_[*u*, *v*] is 1, if *o*_*i*_ (i.e., the perturbed location pair $\left(g_{cur\_i}^{\prime },g_{nex\_i}^{\prime }\right)$) is randomly generated from the true location (*g*_*u*_, *g*_*v*_) by GeoInd. Otherwise, *z*_*i*_[*u*, *v*] is set to 0. For simplicity, we use *s*_*j*,*k*_ to represent the true location pair (*g*_*j*_, *g*_*k*_).

Given the set of observed variables *O* and the set of latent variables *Z*, the complete data likelihood function is defined as follows:

$$\begin{array}{c} \begin{array}{cc} & L(\theta ;O,Z)=P(O,Z|\theta )=P(O|Z,\theta )P(Z|\theta )\\ & =\prod_{i=1}^{\left|DB\right|}P(o_{i}|z_{i},\theta )\times \prod_{i=1}^{\left|DB\right|}P(z_{i}|\theta )\\ & =\prod_{i=1}^{\left|DB\right|}\prod_{j=1}^{mn}\prod_{k=1}^{mn}P{\left(o_{i}|s_{j,k}\right)}^{z_{i}\left[j,k\right]}\times \prod_{i=1}^{\left|DB\right|}\prod_{j=1}^{mn}\prod_{k=1}^{mn}{\pi_{j,k}}^{z_{i}\left[j,k\right]} \end{array} \end{array}$$ (5)

Here, *π*_*j*,*k*_ represents the probability that the current and next locations of a user are *g*_*j*_ and *g*_*k*_ respectively that we aim to estimate by using EM. That is, *π*_*j*,*k*_ corresponds to *P*(*g*_*j*_, *g*_*k*_). Obviously, $\sum_{j=1}^{mn}\sum_{k=1}^{mn}\pi_{j,k}$ equals to 1.

Additionally, *P*(*o*_*i*_|*s*_*j*,*k*_) corresponds to $P(\left(g_{cur\_i}^{\prime },g_{nex\_i}^{\prime }\right)|\left(g_{j},g_{k}\right))$. That is, *P*(*o*_*i*_|*s*_*j*,*k*_) denotes that the perturbed location pair $\left(g_{cur\_i}^{\prime },g_{nex\_i}^{\prime }\right)$ is randomly generated from the true location pair (*g*_*j*_, *g*_*k*_) by GeoInd. We note that the perturbation process of GeoInd is independently applied to each true location in a trajectory. Hence, *P*(*o*_*i*_|*s*_*j*,*k*_) can be computed as follows:

$$\begin{array}{c} \begin{array}{cc} P(o_{i}|s_{j,k}) & =P\left(\left(g_{cur\_i}^{\prime },g_{nex\_i}^{\prime }\right)|\left(g_{j},g_{k}\right)\right)=P\left(g_{cur\_i}^{\prime }\;|\;g_{j}\right)\times P\left(g_{nex\_i}^{\prime }\;|\;g_{k}\right)\\ & =OM[j,cur\_i]\times OM[k,nex\_i] \end{array} \end{array}$$ (6)

Note that by the definition of the obfuscation matrix, *OM*[*j*, *cur*_*i*] equals to $P(g_{cur\_i}^{\prime }|g_{j})$.

Given the Eq (5), the log-likelihood function is defined as follows:

$$\begin{array}{c} \begin{array}{cc} \text{log}L(\theta ;O,Z) & =\sum_{i=1}^{\left|DB\right|}\sum_{j=1}^{mn}\sum_{k=1}^{mn}(z_{i}\left[j,k\right]\text{log}P\left(o_{i}|s_{j,k}\right)+z_{i}\left[j,k\right]\text{log}\pi_{j,k})\\ & =\sum_{i=1}^{\left|DB\right|}\sum_{j=1}^{mn}\sum_{k=1}^{mn}(z_{i}\left[j,k\right]\times (\text{log}\pi_{j,k}+\text{log}\left(OM\left[j,cur\_i\right]\times OM\left[k,nex\_i\right]\right))) \end{array} \end{array}$$ (7)

In the last line of Eq (7), *P*(*o*_*i*_|*s*_*j*,*k*_) is substituted by Eq (6). We now define the method that computes the transition probabilities using the EM algorithm.

#### Initialization

In the initialized phase, the initial parameter *θ*^(0)^ is defined. Determining good initial values in EM is vital to reducing the number of iterative steps until convergence and enhancing the accuracy of parameter estimation. In this paper, we use two different schemes to determine initial values:

- *Uniform initialization:* In the first scheme, *π*_*j*,*k*_ is initialized with a uniform value, such as $\pi_{j,k}=\frac{1}{{\left(m\times n\right)}^{2}}$, which satisfies $\sum_{j=1}^{mn}\sum_{k=1}^{mn}\pi_{j,k}=1$.
- *Distance-based initialization:* Intuitively, as the distance between two locations decreases, the transition probability between them increases. Accordingly, in the second scheme, *π*_*j*,*k*_ is initialized with a value that is inversely proportional to the distance between *g*_*j*_ and *g*_*k*_ (i.e., $\pi_{j,k}\propto \frac{1}{d(g_{j},g_{k})}$), while satisfying $\sum_{j=1}^{mn}\sum_{k=1}^{mn}\pi_{j,k}=1$.

#### E-Step

In this step, the conditional expectation of the latent variables *Z* is estimated based on the current parameter *θ*^(*h*)^. The E-step is stated as follows:

$$\begin{array}{c} \begin{array}{cc} Q(\theta |\theta^{\left(h\right)}) & =E_{Z|O,\theta^{\left(h\right)}}\left[\text{log}L\left(\theta ;O,Z\right)\right]\\ & =\sum_{i=1}^{\left|DB\right|}\sum_{j=1}^{mn}\sum_{k=1}^{mn}(\tau_{i,[j,k]}^{\left(h\right)}\times (\text{log}\pi_{j,k}+\text{log}\left(OM\left[j,cur\_i\right]\times OM\left[k,nex\_i\right]\right))) \end{array} \end{array}$$ (8)

Here, $\tau_{i,[j,k]}^{\left(h\right)}$ can be viewed as a posterior probability that given the current parameter *θ*^(*h*)^, the perturbed location pair $\left(g_{cur\_i}^{\prime },g_{nex\_i}^{\prime }\right)$ is generated from the true location pair (*g*_*j*_, *g*_*k*_). Thus, it can be computed by Bayes’ theorem as follows:

$$\begin{array}{c} \begin{array}{cc} \tau_{i,[j,k]}^{\left(h\right)} & =P\left(s_{j,k}|o_{i},\theta^{\left(h\right)}\right)=P\left(\left(g_{j},g_{k}\right)|\left(g_{cur\_i}^{\prime },g_{nex\_i}^{\prime }\right),\theta^{\left(h\right)}\right)\\ & =\frac{P\left(\left(g_{j},g_{k}\right)|\theta^{\left(h\right)}\right)\times P\left(\left(g_{cur\_i}^{\prime },g_{nex\_i}^{\prime }\right)|\left(g_{j},g_{k}\right),\theta^{\left(h\right)}\right)}{P(\left(g_{cur\_i}^{\prime },g_{nex\_i}^{\prime }\right)|\theta^{\left(h\right)})}\\ & =\frac{P\left(\left(g_{j},g_{k}\right)|\theta^{\left(h\right)}\right)\times P\left(\left(g_{cur\_i}^{\prime },g_{nex\_i}^{\prime }\right)|\left(g_{j},g_{k}\right),\theta^{\left(h\right)}\right)}{\sum_{g_{u},g_{v}\in G}P\left(\left(g_{u},g_{v}\right)|\theta^{\left(h\right)}\right)\times P\left(\left(g_{cur\_i}^{\prime },g_{nex\_i}^{\prime }\right)|\left(g_{u},g_{v}\right),\theta^{\left(h\right)}\right)}\\ & =\frac{\pi_{j,k}^{\left(h\right)}\times OM\left[j,cur\_i\right]\times OM\left[k,nex\_i\right]}{\sum_{g_{u},g_{v}\in G}\pi_{u,v}^{\left(h\right)}\times OM\left[u,cur\_i\right]\times OM\left[v,nex\_i\right]} \end{array} \end{array}$$ (9)

Note that the last line of Eq (9) is rewritten using Eq (6).

#### M-Step

This step finds the parameters *θ* that maximize the expectation function, *Q*(*θ*|*θ*^(*h*)^), in the E-step. This task can be viewed to find the extreme value of Eq (8) with respect to the constraint $\sum_{j=1}^{mn}\sum_{k=1}^{mn}\pi_{j,k}=1$. Therefore, we exploit the Lagrange multiplier method by defining the Lagrange function as follows:

$$\begin{array}{c} L\left(\theta ,\pi ,\mu \right)=Q\left(\theta |\theta^{\left(h\right)}\right)+\lambda \left(\sum_{j=1}^{mn}\sum_{k=1}^{mn}\pi_{j,k}-1\right) \end{array}$$ (10)

Then, the first-order partial derivative of L(θ,π,μ) with respect to *π*_*j*,*k*_ is obtained as follows:

$$\begin{array}{c} \begin{array}{cc} & \frac{\partial L}{\partial \pi_{j,k}}=\frac{\sum_{i=1}^{\left|DB\right|}\tau_{i,[j,k]}^{\left(h\right)}}{\pi_{j,k}}+\lambda =0\Leftrightarrow \pi_{j,k}=\frac{\sum_{i=1}^{\left|DB\right|}\tau_{i,[j,k]}^{\left(h\right)}}{-\lambda } \end{array} \end{array}$$ (11)

By utilizing the constraint to get the optimal value, we have the following:

$$\begin{array}{c} \begin{array}{cccc} & \sum_{j=1}^{mn}\sum_{k=1}^{mn}\pi_{j,k}=1\Leftrightarrow & \sum_{i=1}^{\left|DB\right|}\sum_{j=1}^{mn}\sum_{k=1}^{mn}\frac{\tau_{i,[j,k]}^{\left(h\right)}}{-\lambda }=1\Leftrightarrow & \lambda =-|DB| \end{array} \end{array}$$ (12)

According to Eqs (11) and (12), the parameter that maximizes the expectation function, *Q*(*θ*|*θ*^(*h*)^), is defined as follows:

$$\begin{array}{c} \pi_{j,k}=\frac{\sum_{i=1}^{\left|DB\right|}\tau_{i,[j,k]}^{\left(h\right)}}{\left|DB\right|} \end{array}$$ (13)

Thus, we can obtain the updated parameter $\pi_{j,k}^{\left(h+1\right)}$ using Eq (13), in which $\tau_{i,[j,k]}^{\left(h\right)}$ is already computed in the previous E-step.

#### Computation of transition probability

The abovementioned E-step and M-step are repeated until convergence. After computing the parameters using EM, the transition probability from *g*_*x*_ to *g*_*y*_ is computed as follows:

$$\begin{array}{c} p\left(g_{x}\to g_{y}\right)=\frac{P(g_{x},g_{y})}{\sum_{g_{z}\in G}P(g_{x},g_{z})}=\frac{\pi_{x,y}}{\sum_{g_{z}\in G}\pi_{x,z}} \end{array}$$ (14)

We note that the parameter *π*_*x*,*y*_ is already estimated in the previous E-Step and M-Step.

## Experiments

In this section, we present the experiments we carried out to evaluate the proposed approach. First we describe the experimental setup and then we will discuss the experimental results.

### Experimental setup

We evaluate the proposed method using the T-Drive dataset [20] that contains one-week trajectories of Beijing taxis. We first extract 85707 trajectories, with a length of 10, from the T-Drive dataset. Then, the entire geographic region, represented by longitude and latitude information, is segmented into three different grids: 10 × 10, 15 × 15, and 20 × 20. Each location in trajectories is assigned to a segmented region (i.e., grid) to which it geographically belongs. In the experiments, results are reported for the following alternatives, the straightforward approach (*SA*), the proposed EM-based approach with the uniform initialization (*EM*_*uni*_), and the proposed EM-based approach with the distance-based initialization (*EM*_*dis*_). Furthermore, we compare our proposed method with the particle filter-based approach (*PF*) which has been extensively used in trajectory detection [21, 22]. For our evaluations, we adapted the underlying particle filter-based approach technique for trajectory detection to infer users’ true locations from perturbed locations. Especially, at each iteration of the particle filter, the weights of particles are updated by using the probabilities embedded in the obfuscation matrix *OM* of GeoInd.

To compare these four schemes, we use the mean absolute error (MAE), which is defined as follows:

$$\begin{array}{c} \begin{array}{cc} & MAE=\frac{1}{{\left(mn\right)}^{2}}\times \sum_{x=1}^{mn}\sum_{y=1}^{mn}|p\left(g_{x}\to g_{y}\right)-p^{est}\left(g_{x}\to g_{y}\right)| \end{array} \end{array}$$ (15)

Here, *p*^*est*^(*g*_*x*_ → *g*_*y*_) is the transition probability estimated based on the perturbed trajectory dataset, whereas *p*(*g*_*x*_ → *g*_*y*_) is the true transition probability computed based on the actual trajectory dataset.

### Results and discussion

**Fig 2:**
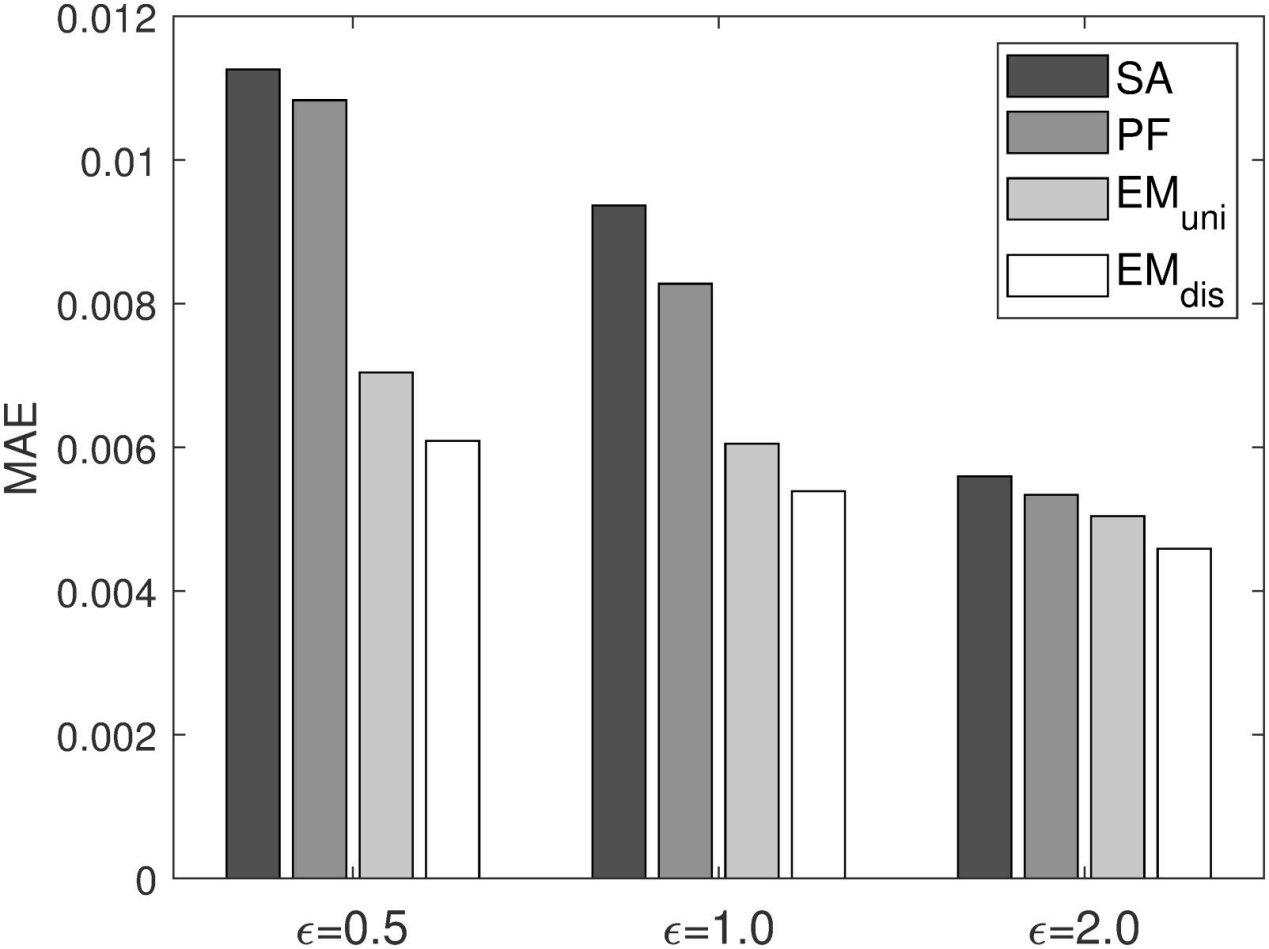
MAE on varying privacy budgets.

Fig 2 illustrates MAE values versus a varying privacy budget *ϵ*. In the experiments, *ϵ* varies from 0.5 to 2.0, while the size of grid is fixed to 10 × 10. Key observations based on Fig 2 can be summarized as follows: As expected, the error rate increases as the privacy budget decreases. This is because a smaller *ϵ* value provides stronger privacy. However, in terms of utility, a smaller *ϵ* results in a lower utility of the collected trajectory dataset. This in turn leads to a decreased estimation accuracy when computing the transition probability. On the contrary, a larger value of *ϵ* provides a weaker privacy guarantee, while introducing less perturbation to true locations. This in turn leads to an increased estimation accuracy of the transition probability.

Among the four alternatives, the proposed EM-based schemes, *EM*_*uni*_ and *EM*_*dis*_, exhibit the better performance compared to the straightforward approach *SA* and the particle filter-based approach *PF*. Especially, the performance gain of the proposed EM-based schemes over *SA* and *PF* is more pronounced at a smaller *ϵ* value, which provides stronger privacy. Considering that many real-world applications require stronger privacy protection guarantees against attackers with arbitrary backgrounds, these results indicate that the proposed method is more practical for real-world applications. Between the two EM-based schemes, *EM*_*dis*_ slightly outperforms *EM*_*uni*_ at all privacy bugets, which indicates that the distance-based initialization scheme is more promising than a uniform value scheme.

**Fig 3:**
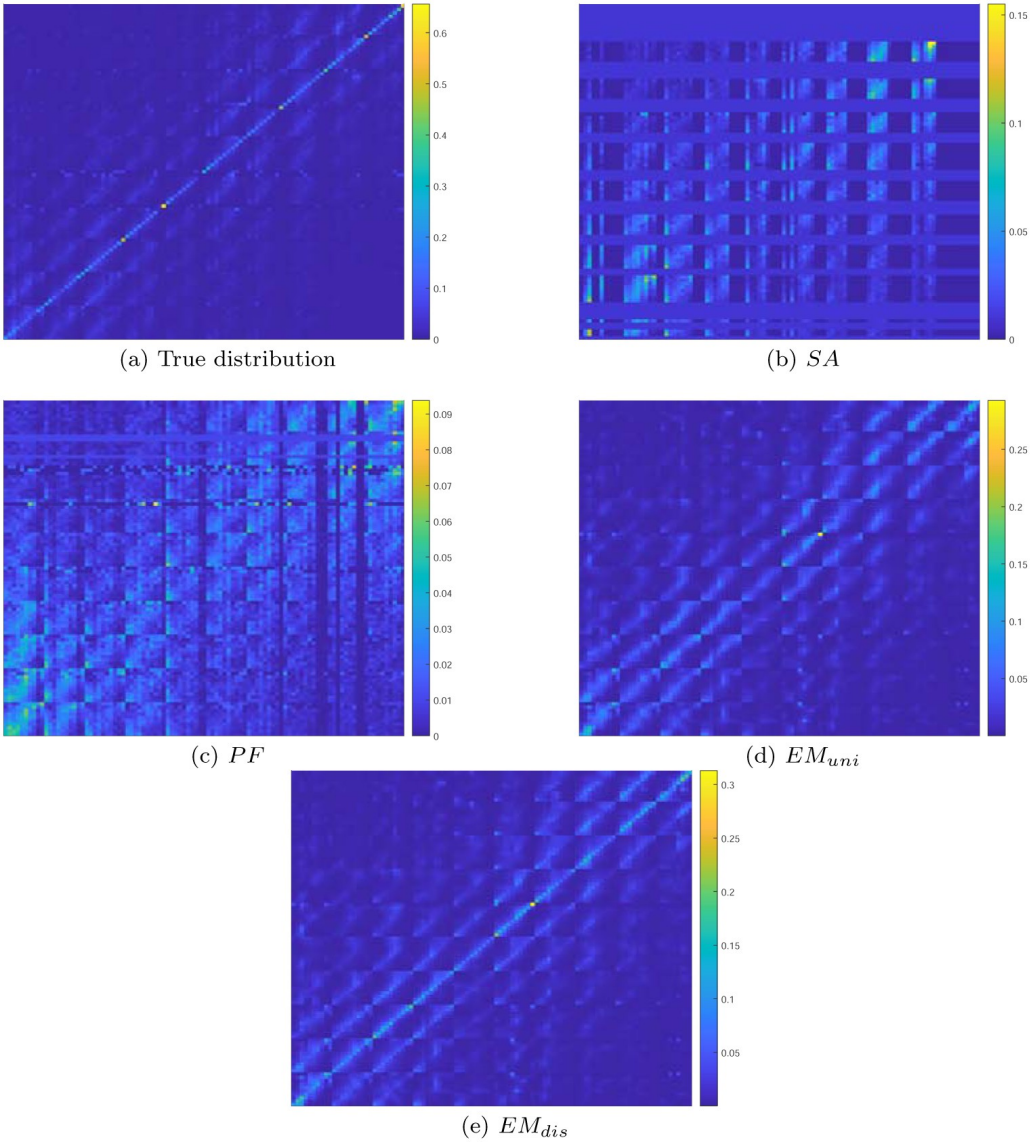
Heatmaps for the transition probability distribution.

To further investigate the validity of the experimental results, in Fig 3, we plot a heatmap for the estimated transition probability distribution of each method with *ϵ* being set to 1.0. The size of grid is set to 10 × 10. For comparison purposes, we also plot the true transition probability distribution that is obtained with the actual trajectory dataset. As observed in the figure, the estimated probability distribution obtained by *SA* and *PF* is quite dissimilar to the true distribution. On the contrary, with the proposed EM-based schemes, we can obtain the estimated probability distributions that are highly similar to the true one. Between the two EM-based schemes, the estimated probability distributions obtained by *EM*_*dis*_ are more similar with the true distributions than those computed by *EM*_*uni*_. The experimental results presented in Figs 2 and 3 indicate that the proposed EM-based approach can achieve higher precision in the computation of the transition probability with trajectory dataset collected in a privacy-preserving manner using GeoInd than that of the straightforward and particle filter-based approaches.

**Fig 4:**
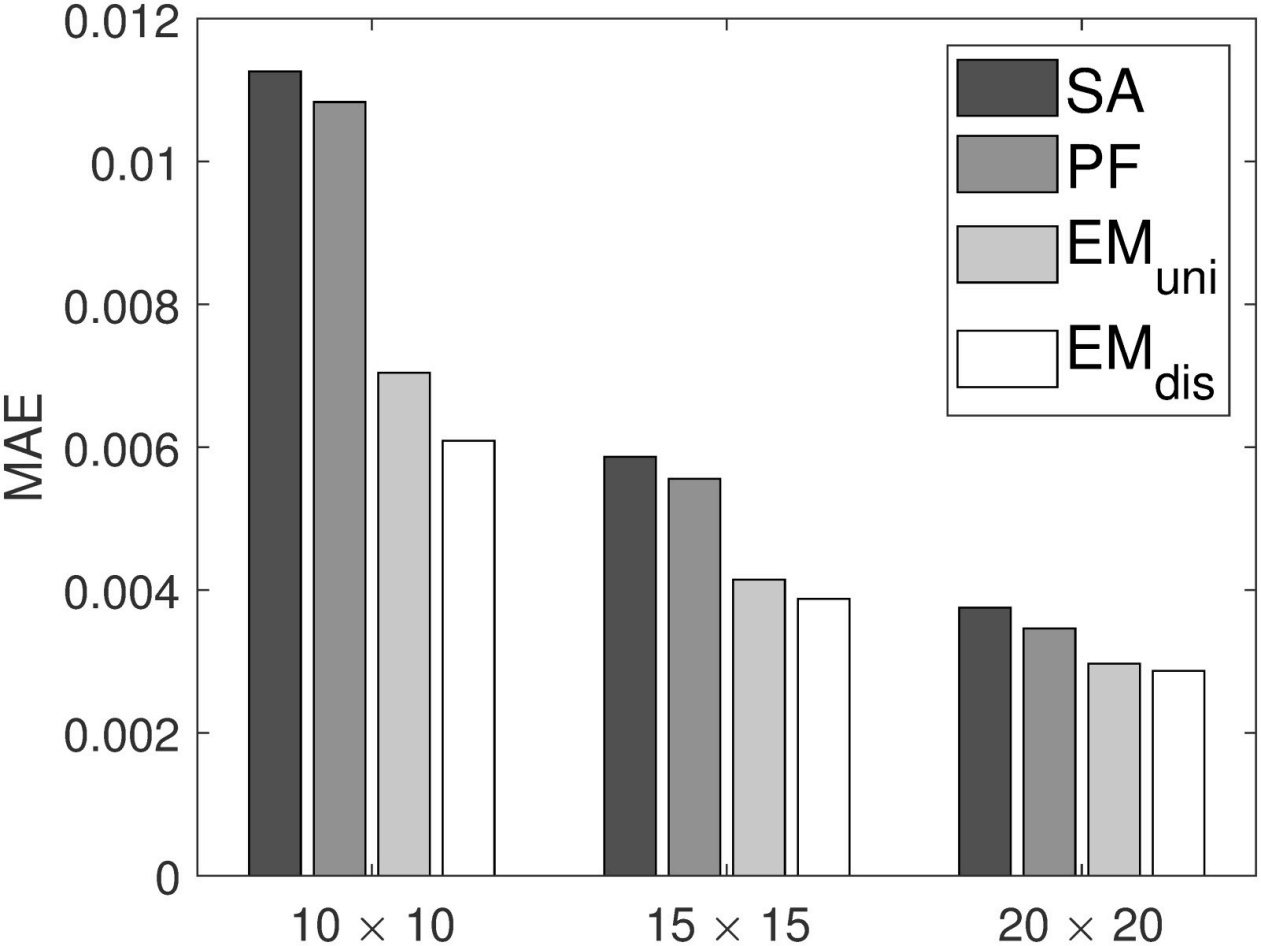
MAE on varying grid sizes.

Fig 4 shows MAE values with respect to various grid sizes. In the experiment, three different grid sizes are used with *ϵ* fixed to 0.5. Similar experimental results can be observed with various grid sizes. As shown in Fig 4, for all grid sizes, the proposed *EM*_*uni*_ and *EM*_*dis*_ significantly outperform *SA* and *PF* regarding MAE, verifying the robustness of the proposed method against the grid size. Furthermore, the EM scheme using the distance-based initialization achieves slightly better performance than that using the uniform-based initialization.

**Fig 5:**
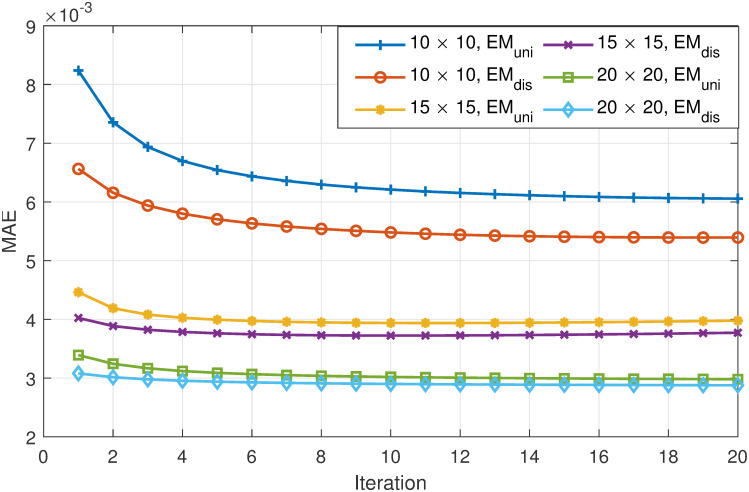
MAE vs. the number of iterations of EM.

Finally, we validate the convergence of the proposed EM-based schemes. In Fig 5, we plot the MAE values versus the number of iterations in the EM process. In the experiments, *ϵ* is fixed to 1.0, while three different grid sizes are used. As shown in the figure, we can observe a significant drop in the MAE values during the first a few iteration. Beyond this point, the MAE values stabilize. These experiment results verify that for the proposed EM-based scheme that computes the transition probability, the number of iterations guaranteeing convergence is small. This in turn indicates that the additional computational overhead of the proposed scheme incurred when using the EM algorithm is not significant.

## Related work

GeoInd have been used in diverse application domains. Here, we present some application areas of GeoInd. Vehicle networks have recently emerged as a promising solution to improve driving experiences and road safety. In [23], Zhou *et al.* proposed edge-assisted vehicle networks for improving the service quality in the vehicle networks where GeoInd is deployed at the edge nodes to protect the true location of the vehicle. In the proposed system, a vehicle first submits a service request along with its location to an edge node at which the GeoInd-based mechanism is executed to protect the actual location of the vehicle, and the service request with the perturbed vehicle’s location is then forwarded to the service provider which returns the required service to the requesting vehicle. To protect location privacy in location-aware social networks, GeoInd combined with homomorphic encryption is used for privacy-preserving nearby friend discovery [24]. Spatial crowdsourcing is a platform where individual users are engaged to collect, analyze, and disseminate their surrounding information. Wang *et al.* [16] proposed a differential geo-obfuscation mechanism to protect the workers’ true location during task assignment by an spatial crowdsourcing platform. Yan *et al*. [25] developed a spatial crowdsourcing framework which can protect the privacy of the workers’ trajectories. In the developed framework, the GeoInd mechanism is used to protect a worker’s shortest path from the source to destination. Qui *et al*. [17] investigated location-privacy protection in a vehicle-based spatial crowdsourcing framework using GeoInd. To address the location privacy issues raised in ride-sharing applications such as Uber, Waze, and Lyft, Tong *et al.* [26] proposed a scheduling scheme which exploits GeoInd to protect the location information of ride-sharing users. We note that the existing works fucus on either protecting users’ location privacy using GeoInd or extracting static information, such as population density distribution, from perturbed location datasets collected under GeoInd setting. On the contrary, the objective of this work is to estimate users’ moving information, such as transition patterns, with perturbed trajectory datasets collected using GeoInd.

There have been extensive studies to leverage the concept of differential privacy for publishing trajectory data in a privacy-preserving manner. Hua *et al*. [27] proposed a differential-privacy-based scheme for publishing time-serial trajectory data. The proposed scheme leverages an exponential mechanism to probabilistically cluster locations based on their distance, and then relies on the Laplace mechanism to add a random noise to the count of trajectories in a cluster. DP-Star [28] is a differential-privacy-based framework for publishing trajectory data with strong utility. DP-Star generates synthetic trajectories that satisfy *ϵ*-differential privacy, while maintaining high utility. SafePath [29], which is a privacy-preserving algorithm for publishing trajectories, structures trajectories as a noisy prefix tree and publishes differentially-private trajectories, while retaining data utility. Ou *et al*. [30] proposed two lagrange multiplier-based differentially private approaches, UD-LMDP and UC-LMDP, to address privacy issues arising when publishing mutually correlated trajectories. The authors introduced an *n*-body Laplace framework which aims to prevent adversaries from inferring a social relation from the mutual correlation between two users’ trajectories. Chen *et al*. [11] proposed RNN-DP, which is a differential privacy scheme based on a recurrent neural network, to protect the privacy of real-time trajectory data. RNN-DP exploits a recurrent neural network to efficiently predict trajectory, while protecting the location privacy of users using differential privacy. OPTDP [31] is an optimal personalized trajectory differential privacy mechanism for trajectory data publishing. Zhao *et al*. [32] introduced a method to build the SR tree, which is based on R-tree, using the trajectory sequence, and then adds random noise to the nodes in the SR tree. Then, the noise SR-Tree indexes are used to answer user’s spatial queries. Liu *et al*. [33] developed a trajectory data publication scheme that exploits the staircase mechanism of differential privacy.

Differential privacy has been also used to process and analyze trajectory data in diverse application areas. PLDP-TD [34] supports personalized-location differentially private data analysis on trajectory databases. To answer queries in a differentially private way, PLDP-TD builds a personalized noisy trajectory tree which stores sub-trajectories of a trajectory database along with their privacy protection levels. That is, a personalized privacy level is assigned to each node of the tree depending on the privacy protection requirements of locations. Kim *et al*. [35] leverages local differential privacy, which is a localized version of differential privacy, to collect indoor positioning data from users, while protecting their location privacy. ConCrowd-DP [36] is a differentially private framework for mobile crowdsourcing applications in which the mobile users upload the location-related task results to the server for obtaining the rewards. In ConCrowd-DP, to protect of participating users’ location privacy, perturbed locations are used instead of users’ true locations, when reporting the location-related task results to the server. Deldar and Abadi [37] proposed DPLG to efficiently and accurately answer spatial queries on moving objects databases. DPLG is based on the combination of differential privacy and location generalization which enable to efficiently and accurately process spatial queries by reducing the number of locations and minimizing query errors.

## Conclusion

The most common analysis task in applications that utilize mobility information of users is to compute the transition probability between two consecutive timestamps. Computing transition probabilities from a collection of privacy-preserved trajectories is challenging, because of a loss in the utility of collected dataset caused by a privacy-preserving mechanism. To address this challenge, in this paper, we proposed a novel scheme to compute the transition probability with the collection of perturbed trajectories collected using GeoInd. The proposed method leveraged the EM algorithm to precisely estimate hidden transition patterns based on a perturbed trajectory dataset. Experimental results with real datasets verified that a good estimation of transition pattern can be achieved with the proposed method.
